# Using a Blended Approach to Thematic Analysis: A Case Study on Fatherhood and Imprisonment

**DOI:** 10.1093/poq/nfaf033

**Published:** 2025-08-06

**Authors:** Marieke Haan, Simon D Venema

**Affiliations:** Assistant Professor, Department of Sociology/ICS, Faculty of Behavioral and Social Sciences, University of Groningen, Groningen, The Netherlands; Researcher, Addiction Care Northern Netherlands, Groningen, The Netherlands; and Researcher, Research Group of Addiction Science and Forensic Care, Hanze University of Applied Sciences, Groningen, The Netherlands

## Abstract

Thematic analysis (TA) has become a prominent qualitative research method for identifying patterns in experiences, responses, narratives, and meanings in relation to a research question. In this paper, we provide researchers with an understanding of different approaches to TA and offer practical guidance on conducting a blended approach to TA, which combines the strengths of different TA approaches. Despite their prevalence in the literature, blended approaches to TA are rarely clearly illustrated in research publications. To address this gap, we present a detailed case study of a blended approach to TA, based on a qualitative study exploring the experiences of incarcerated fathers participating in a prison family program in the Netherlands. This case study offers researchers practical guidance for thoughtfully implementing a blended approach to TA in their own work. It also highlights the nuances and complexities of a blended approach to TA, emphasizing the importance of aligning the chosen TA approach to the research context and objectives. We conclude by discussing the value of blended approaches to TA in qualitative research, underscoring the importance of thoughtful application and transparent reporting of these approaches.

## Introduction

Thematic analysis (TA) has evolved over time to become a prominent approach in qualitative research in the social sciences and beyond. By going through several analytical phases, the researcher aims to identify or construct themes that capture patterns of people’s experiences, responses, narratives, and meanings in relation to a research question (Braun and Clarke 2006, 2012). The end result of conducting a TA is to understand the prominent patterns of meanings found within the dataset (Joffe 2012). TA can be used to analyze a wide variety of qualitative data, including interviews (Cowie and Braun 2022), focus groups (King and Ussher 2013), open-ended questions in surveys (Chen et al. 2018), written documents (Mackieson et al. 2019), observational data (Yukhymenko et al. 2014), and visual data sources (Joffe 2012). TA is widely used in both academic research and applied settings, across various disciplines such as psychology (Hayfield and Wood 2019), sociology (Lewandowska and Smolarska 2020), political sciences (Macq and Jacquet 2023), and health sciences (Sillence et al. 2007) to explore and understand complex phenomena, either solely based on qualitative approaches or complementing quantitative approaches.

In this paper, we offer researchers an understanding of different approaches to TA and provide guidance by presenting a case study for thoughtfully conducting a blended approach to TA in practice. Our primary contribution is to present an example of a blended approach to TA that combines elements of different TA approaches. The case study is about research that was conducted with incarcerated fathers concerning their experiences with an in-prison family program in the Netherlands (Venema et al. 2024).

## Thematic Analysis Approaches

While many researchers cite Braun and Clarke (2006) when using TA in their research (i.e., 220,690 citations on Google Scholar—November 18, 2024), there is not one single standardized form of TA. The lack of awareness or concern of the different approaches to TA has often led to inconsistent and variable use of TA (Braun and Clarke 2019; Stevens 2023). Different forms of TA use distinct analytical procedures, which stem from differences in the researcher’s underlying philosophical assumptions about reality and how it can be known or studied (known as ontological and epistemological orientations). In qualitative research, it is considered good practice to make these assumptions explicit, as they shape the researcher’s approach to data analysis. For example, some TA approaches assume that objective and measurable facts about reality emerge from the data independent of the researcher, similar to underlying assumptions often implicitly made by quantitative researchers. Other TA approaches assume that qualitative research reflects subjective, co-constructed realities shaped by both the participant and the researcher. Therefore, for every researcher considering TA, it is important to understand what type of TA suits the researcher’s assumptions about reality and how it is studied (Braun and Clarke 2023a, 2023b). For a more detailed discussion of the role of philosophical foundations in TA, we refer to the work of Braun and Clarke (2022).

In the following section, we briefly discuss various perspectives on TA approaches using the three approaches constructed by Braun and Clarke (2022): coding reliability approaches, codebook approaches, and reflexive approaches. Then, we discuss the use of blended TA approaches.

### Three Approaches to Thematic Analysis

First, *coding reliability approaches* aim to objectively understand reality by identifying patterns, facts, or causes that are assumed to emerge from qualitative data through appropriate scientific methods. However, it is also recognized that the acquired knowledge will never be completely perfect or unbiased (Braun and Clarke 2022). The work of Boyatzis (1998) and Guest et al. (2012) can be considered as TA coding reliability approaches. These approaches are characterized by early and often pre-identified (i.e., deductive) code and theme development. The pre-identified codes are then placed together in a codebook. The codebook acts as a predefined guide for researchers, providing a systematic structure for organizing and categorizing data, ensuring consistency and coherence throughout the analysis process. To ensure objective and accurate coding, an integral part of coding reliability approaches is demonstrating consensus among researchers through intercoder reliability checks. Consensusbuilding is prioritized to strengthen the robustness and validity of themes, thereby minimizing subjectivity and individual biases (see Joffe 2012 for further reading).

Second, TA *codebook approaches* are flexible to accommodate different ways of thinking about TA research. Codebook approaches can either assume that there is an independent, objective reality that can be observed through thorough analysis, or that reality is subjective and mediated through language and culture, and that knowledge of this reality is actively co-constructed by both the researcher and the researched. Examples of TA codebook approaches include framework analysis (Ritchie and Spencer 1994) and template analysis (King 1998; King and Brooks 2018; based on Crabtree and Miller’s early work in 1999). Additionally, using data matrices for analyzing qualitative data (Nadin and Cassell 2004) and thematic network analysis (Attride-Stirling 2001) can also be seen as TA codebook approaches (Braun and Clarke 2022). Although codebook approaches often rest on a predefined framework or template for coding, they are more flexible, not as rigidly structured as coding reliability TA. This is because the codebook approach allows for themes to be predefined at an early stage of the analysis and also constructed as the analysis progresses and new insights are developed, allowing for nuanced interpretations within the analysis. Themes identified through codebook approaches may be translated into topic summaries, aiming to summarize the essence of the data in a coherent and meaningful way, or as shared meanings, reflecting a more interpretive orientation toward thematic development as in reflexive TA approaches (Connelly and Peltzer 2016; see also Braun and Clarke 2022). Reflexivity, the critical self-awareness of the researcher’s role and influence in the research process, is important in codebook approaches (see Parkinson et al. 2016 for a TA codebook approach example).

Third, *reflexive approaches*, as advocated by Braun and Clarke (2019, 2022, 2024), aim to understand how meanings and realities are constructed by individuals, rather than seeking objective knowledge. Reflexivity is central to this approach, meaning that researchers actively acknowledge the role of their own position, perspectives, and presence in the collection, selection, and interpretation of data (Finlay 2002; Lazard and McAvoy 2020). Critical to reflexive approaches is the deep appreciation for the personal perspectives and experiences of the researcher and acknowledging how these can influence the research process. This is addressed by emphasizing the researcher’s active role in interpreting and constructing themes from the data and by encouraging reflexivity throughout the process, such as keeping reflective journals or engaging in discussions with other researchers about these personal perspectives and experiences. Furthermore, in contrast to structured coding procedures that typically rely on predefined codes (as in coding reliability approaches), reflexive approaches involve an organic coding process. This means that codes are not fixed in advance, but are developed dynamically based on the content of the data itself (Braun and Clarke 2019; Byrne 2022). Researchers continuously review and adjust their codes throughout the analysis, refining them to better capture meaningful patterns and develop themes that represent the underlying essence of the data. This ensures that the analysis reflects the nuance and richness of the data. A defining characteristic of reflexive approaches is also the timing of theme development, which often occurs at a later stage of the analysis. The themes are actively constructed by the researcher from the developed codes, either inductively based on the information from the data itself and/or deductively when taking theoretical knowledge into account when coding the data (Braun and Clarke 2019). Unlike in coding reliability and codebook approaches, themes are conceptualized as collective interpretations and understandings that transcend individual experiences, highlighting patterns of shared meanings within the data (see Byrne 2022 for an example of reflexive TA).

### Blended Approaches

Following the stages of a particular TA approach can be helpful for researchers in structuring the analysis process. Stevens (2023) refers to this as an “orthodox approach” to qualitative research and further argues that a pragmatic mindset is essential to ensure that the data analysis fits the research question, is connected to the direction of the research, and ensures high-quality output. Although the three TA approaches outlined above can be used in many situations, they can also be blended. We use the term “blended approach” to refer to TA approaches that combine the strengths of different approaches and to overcome the limitations of a single approach. The idea of blending approaches fits well with the work of Finlay (2021), who argues that TA researchers value both scientific rigor and artistic expression. This means that on the one hand, TA researchers often attempt to systematically code and categorize the data, which is consistent with coding reliability and codebook approaches. On the other hand, TA researchers seek to ensure that the analysis is subject to reflexivity and creatively presented, which is more consistent with reflexive approaches and some codebook approaches. Although blended approaches are commonly used to conduct qualitative research analysis, Braun and Clarke (2021) note that these blended approaches are not often clearly discussed in scientific work using TA. They emphasize that while blended approaches (or “mash ups”) are not inherently problematic, they often lead to theoretical and conceptual incoherence if not carefully considered and reflected upon. In the following section of the paper, we provide a case study focusing on fatherhood and imprisonment in the Netherlands (Venema et al. 2024) to illustrate how a blended approach of codebook and reflexive TA can work in practice when thoughtfully executed.

## Case Study: Using a Blended Approach to Thematic Analysis to Study Imprisoned Fathers’ Experiences of an In-Prison Family Program

### Outline of the Study

The purpose of the research project was to examine imprisoned fathers’ experiences of an in-prison family-focused program in a prison in the Netherlands. The primary goals of the program were to (1) reduce negative impact of paternal imprisonment on children, (2) stimulate positive family relationships, (3) reduce fathers’ recidivism after release, and (4) reduce intergenerational transmission of criminal behavior.

The study was designed relatively broadly, encompassing a wide variety of topics related to fatherhood, family relationships, children’s well-being, and imprisonment. A unique feature of this study was that we compared fathers’ experiences who participated in the program with a comparison group of fathers in prison who did not participate in the program. Although relatively uncommon in research using TA, using a comparison group in qualitative evaluation research allowed us to identify key differences between the experiences of participants and nonparticipants. This approach helped pinpoint the primary mechanisms driving the differences between the two groups (Lindsay 2019).

We used a blended approach to TA, containing elements of both codebook and reflexive approaches, which allowed us to provide a nuanced analysis. This blended approach aligned with our thinking about qualitative research, as we assumed that reality could be studied through participants’ experiences and perspectives, and that knowledge about this was co-constructed by both the participants and the research team.

The research team was composed of Dutch researchers from the University of Groningen and the Hanze University of Applied Sciences Groningen. The team possessed extensive expertise in qualitative research, as well as in topics related to sociology and criminology. Three members of the research team were co-involved in the development of the family approach program and thus had in-depth knowledge of the research context and the population of interest (though they were not involved in the implementation and day-to-day activities of the program).

### Data Collection

We collected data in three prisons between November 16, 2021, and March 21, 2022, using a purposive sample containing 39 semistructured interviews with imprisoned fathers (*N *= 10 fathers in the family program group, *N *= 29 fathers in the comparison group). The population consisted of fathers in detention who maintained contact with their children during the detention period. For the data collection, an interview guide was developed based on a previous systematic review on fatherhood in prison (see Supplementary Material and Venema et al. 2022 for more details on the literature review; for the full interview guide, see Venema et al. 2024). All interviews were conducted face-to-face and audio-recorded, transcribed verbatim, and anonymized during transcription. Participants were assured of anonymity and confidentiality and provided informed consent. The study was approved by the Hanze University of Applied Sciences Groningen Ethical Review Board (reference number: heac.2021.031a). For more details about the data collection, see Venema et al. (2024).

### Data Analysis

The aim of the TA was to construct themes that captured differences in experiences, responses, narratives, and meanings between fathers participating in the family program and those in the comparison group. Data were coded using a combination of deductive and inductive coding. Our blended strategy to TA included elements of both codebook and reflexive approaches. In retrospect, while we initially referred to this as a “codebook” approach in the original article (Venema et al. 2024), further reflection and analysis indicated that our TA more accurately represents a blended approach combining structured and reflexive elements. Considering elements of the codebook approach, we developed a codebook of predefined codes based on systematic review (Venema et al. 2022) that could be used consistently by all members of the research team. Throughout the analyses, we worked continuously to build consensus among the research team about these codes in order to interpret and use the codes in a similar way.

Our approach also included key elements of reflexive TA approaches. Specifically, we adhered to the stages of analysis outlined by Braun and Clarke that facilitate alignment with reflexive TA (2022, 2024). This is important to mention because, unlike other TA approaches such as framework analysis or template analysis, each of which has its own set of stages, following Braun and Clarke’s stages facilitates alignment with reflexive TA. Furthermore, our data analysis process was organic and circular, meaning we reviewed and adjusted our codes continuously and these new insights were integrated into the codebook as our analysis progressed (Byrne 2022). Throughout the analysis, the theoretical framework and research question were refined as well. We also took time to reflect on our own perspectives regarding the topic under investigation (fatherhood and imprisonment). This was achieved through team meetings and by maintaining a research journal in which analytical ideas, field notes, decisions, and reflections were recorded. This strategy aligns with the recommendations of Braun and Clarke (2023a) and Levitt et al. (2017) for ensuring quality in qualitative research.

### Discussion of Phases

We use the six-phase model of TA to structure our discussion of the TA case study (Braun and Clarke 2022, 2024). This includes the following six phases: (1) familiarizing with the dataset, (2) coding, (3) generating initial themes, (4) developing and reviewing themes, (5) refining, defining, and naming themes, and (6) writing up. Although these six phases appear to be linear, our process involved moving back and forth between them in an iterative, organic way (also see Saldana 2021).

#### Phase 1: Familiarizing with the dataset

Data familiarization started during data collection. In line with a more reflexive approach to TA, we organized meetings in which we reflected on our experiences of the interviews. In this phase, we developed first analytical insights about the differences between fathers in the family program and those that were not. Furthermore, we reflected on other experiences that would be difficult to capture in the transcripts. Such reflections dealt with issues such as participants’ nonverbal communication, the participants’ attitudes, the setting in which the interview took place, and topics relating to social desirability. Recurring topics of discussion included social and cultural differences between interviewers and participants regarding family and parenting, and our thoughts about social desirability in relation to participants’ portrayals of their roles in the family. Data familiarization progressed during the transcription of the interviews, which involved listening to all recorded interviews, which allowed us to take into account subtle differences in participants’ tone, such as sarcasm and irony. In this phase, we also wrote down first analytical ideas for inductive codes. In addition, we wrote meta-comments that included reflections of the entirety of the dataset rather than the individual interviews. An example of a meta-comment that we noted is that fathers often stated being highly involved in their children’s lives before imprisonment yet many fathers found it difficult to discuss their day-to-day involvement in detail. These meta-comments were reflected upon in research team meetings, and proved useful in writing the discussion section of the manuscript.

#### Phase 2: Coding

The codes were developed on the basis of (a) theoretical insights and existing literature, and (b) analytical ideas developed during data familiarization. In line with codebook approaches, we started with a list of predefined deductive codes based on our systematic literature review (Venema et al. 2022) and placed these in a codebook. We chose to develop codes that were mostly descriptive in nature rather than interpretative, as this more readily facilitated coding in a team. The codebook was developed by one researcher, then discussed and refined based on discussions within the research team. The codebook was further refined during the coding of the transcripts. A selection of interviews was doublecoded by two different researchers, which were then compared and discussed and led to a new consensus of code descriptions. The purpose was not to ensure intercoder reliability, but to clarify our collective interpretation behind the use of each code. We then developed definitions and descriptions of codes to ensure that the codes would be used in similar ways by different members of the research team. After finishing the codebook, we used the qualitative data analysis program ATLAS.ti 22 for Windows to code the data. The interview transcripts were coded by two researchers in the team, and this process was monitored by a third researcher. In the final stage, differences in code use between the two coders were identified and compared, then taken into account in the subsequent analysis. The final codebook contained 55 codes consisting of deductive as well as inductive codes. In line with a reflexive approach, we reflected upon the role of the researcher throughout the entire process.

#### Phase 3: Generating initial themes

After the full dataset was coded, we analyzed, categorized, connected, and interpreted the codes across the dataset as a whole and between the family program group and the comparison group. To start, we inspected differences in the relative frequency of code use between the two groups to obtain a first idea of differences and similarities across the two groups. Although the frequency of code use does not necessarily equate to the importance of a code, the differences in frequency did provide a first indication of where differences between the two groups could be identified. Then, we inspected which codes were commonly used in tandem. This allowed us to develop ideas about which codes were used in which context. Next, we studied the content of the coded data, and made comparisons between fathers participating in the family program and those in the comparison group. Additionally, based on the coded data, two members of the research team independently developed a list of the most important broader preliminary themes in the data, which were then compared and discussed to inform the analyses.

As the analysis progressed, we found that the most salient patterns could be identified across three outcome domains: father involvement, fathering role, and quality father-child relationships. We therefore decided to focus the analysis on these outcomes, which were also closely aligned with the in-prison family-focused program’s goals. This flexibility in research focus, informed by insights gained during data analysis, can be considered an element of the reflexive component of the blended approach to TA. With this renewed focus, we studied the content of the coded data, and made comparisons between fathers participating in the family program and those in the comparison group. The aim was to look for *patterned* differences in the experiences, responses, narratives, and meanings of the two groups. To illustrate, interview segments that were coded as *experiences of family visits* were compared across the family program group and the comparison group. During these comparisons, we searched for differences in the experiences of the two groups, and for underlying mechanisms and processes that explained these differences. Then, we searched for patterns in the differences across groups of codes to inform theme development. As such, we aimed to not only summarize the differences between the two groups (i.e., topic summaries), but also to identify processes that explained these differences (i.e., shared meanings), which is closely aligned with reflexive approaches, but also with some codebook approaches.

#### Phase 4: Developing and reviewing themes

First ideas for potential themes were drafted and continuously refined during the theme development process. Theme construction was an organic process, consistent with a reflexive approach to TA. Looking back, three broad stages of theme construction can be distinguished, as summarized in table 1. The first set of themes was developed relatively early in the analyses (before refining the research question), and involved many subthemes. At this stage, themes were relatively thin and fragmented. In the intermediate stage, the second set of themes was developed after extensive analysis of differences between the coded data segments. While these themes were substantively close to the final set of themes, they were still of a descriptive nature and not sufficiently organized to capture the essence of the theme. Then in the final stage, after recoding this data and multiple discussions within the research team, the third and final set of themes was constructed.

**Table 1.** Stages of theme development.

Phase in theme development	(Preliminary) themes	Comments
1: Early stage theme development	Family contact Family relationships Father involvement Children’s well-being	We initially focused on four themes based on our interview guide and findings from the literature. The purpose of these initial themes was to identify content that was present across the data. We focused on both similarities and differences between the two groups in our study. These initial preliminary themes were largely based on the interview guide, and were constructed during the coding process.
2: Intermediate stage theme development	Facilities for meaningful family contact allow for increased father involvement during imprisonment Fewer perceptions of inability to perform a fathering role from prison Facilities for meaningful family contact may positively affect father-child relationships Realistic perceptions of return to family life after imprisonment Presence of structural barriers limit the potential impact of family program	Based on the insights developed throughout the data analysis, we decided to focus our research question on the differences (rather than similarities) between imprisoned fathers participating in the family program and the comparison group in terms of their experiences of fatherhood. We found that examining the differences between the two groups provided more meaningful insights into the data and a better addition to the existing literature than examining the similarities. We decided to not focus on children’s well-being (theme 4 in early stage theme development), and to focus on father-child relationships rather than family relationships. We found that expectations of return to family life after imprisonment differed across the two groups, which was unexpected beforehand, and constructed a theme based around this finding. The fifth theme was derived from the findings of the negative case analysis. Although these preliminary themes were already more clearly defined than in the early stage theme development, these themes were still rather thinly described. As such, these themes can be seen as an intermediate stage between topic summaries and themes conceptualized as shared meanings. Preliminary names were given to the themes. The primary constructs aligned fairly closely with the themes in the final stage of theme development.
3: Final stage theme development	Differences in positive engagement activities Differences in reflections on fathering role Differences in narratives of how imprisonment affected father-child relationship quality Differences in expectations of family life after imprisonment	Regarding the first theme, we found that it was not father involvement per se that differed between the two groups, but a specific element of father involvement, namely positive engagement activities. We therefore decided to largely focus our first theme on this construct. Regarding the second theme, we focused on fathers’ reflections on their role as fathers, as this was the element in which the two groups differed most substantively. We thus decided to focus our second theme on this particular element. Regarding the third theme, in the final analysis, we realized that it was not the father-child relationships per se that differed between the two groups, but rather the fathers’ narratives of these relationships. Mentioning this term in the name of the theme was thus more accurate. The fifth theme from the intermediate stage of theme development, derived from the negative case analysis, was incorporated into all four final themes. This was done because the findings from the negative cases were relevant to all four themes, but the individual experiences of these cases did not warrant the construction of a separate theme. In addition, we found that the original names of the themes from the intermediate stage theme development were too focused, and we expanded the names of the themes to be more inclusive of the patterns found in the data. In this final stage, the final definitions were given to the themes (i.e., central organizing concepts).

To add rigor to the analyses, we conducted a negative case analysis, meaning we looked for cases that contradicted the main narrative constructed across the themes. This approach allowed us to evaluate alternative explanations and to define the limitations of the research findings, and to develop a richer understanding of our findings (Booth et al. 2013). We searched for cases for whom participation in the family program did not lead to differences in the outcome of interest. To illustrate, the negative case analysis showed that there were two fathers who participated in the family program but did not make use of the family-friendly visiting room or video calling. In contrast to the main narrative of the thematic analysis, these fathers experienced no differences in terms of positive engagement activities relative to the comparison group. As such, the negative case analysis was highly informative of the barriers that diminish the potential impact of in-prison parenting and family relationship programs.

#### Phase 5: Refining, defining, and naming themes

When defining the themes, we aimed to develop themes that were (a) internally organized around a central unifying concept, (b) unique and sufficiently different from each other, and (c) captured a coherent narrative about the overall dataset in relation to the research question (Braun and Clarke 2022; Byrne 2022). For each theme, we aimed to define its core meaning (i.e., the central organizing concept; see also Braun and Clarke 2022). This exercise forced us to critically think about the essence of the theme, and of its boundaries. In this stage, we also checked whether the final themes matched the participants’ narratives by rereading interview transcripts and notes made during data familiarization. The four final themes were: (1) differences in positive engagement activities, (2) differences in reflections on fathering role, (3) differences in narratives of how imprisonment affected father-child relationship quality, and (4) differences in expectations of family life after imprisonment. We decided that relatively descriptive theme names best suited the patterns we constructed in the data, which might be considered more typical of a codebook approach than a reflexive approach to TA. Although these descriptive names may suggest topic summaries consistent with some codebook approaches, the concepts in the themes contained a high degree of researcher interpretation, and a focus on patterns of differences in shared meaning between the two groups centered around a core idea, consistent with a reflexive approach to TA (see also table 1).

#### Phase 6: Writing up

The final phase of producing the report took place in tandem with the fifth phase. While writing, we realized that some themes were more developed than others. As such, we returned to the data to provide more detail for the theme. In this phase, we also selected data extracts that provided evidence for the analytical argument of the theme from a variety of participants. Furthermore, we aimed to discuss the themes in a logical order in the results section.

## Discussion

In this paper, we have discussed different approaches to TA and provided guidance on how to conduct a blended approach to TA in practice. In our blended approach, we sought to combine the strengths of both codebook and reflexive approaches to TA. On the one hand, elements of the codebook approach provided us with structure and consistency, which is beneficial for team collaboration and systematic analysis. On the other hand, elements of the reflexive approach provided flexibility and depth, allowing for a more nuanced and reflective engagement with the data. As such, our blended approach can be seen as positioned between a codebook and a reflexive approach.

### Reflecting on Strengths and Limitations

Our blended approach to TA offered several strengths. By establishing predefined codes, a feature of codebook approaches, we ensured a high degree of structure and consistency in coding across multiple coders, which facilitated collaboration within a team. However, we also allowed for flexibility in both coding and theme development, adapting our research focus based on new findings as the analyses progressed, which is more typically associated with reflexive TA. This adaptability was critical, as more rigid approaches may limit the incorporation of new findings as the analysis progresses. The value of this flexibility is evident in our process of developing themes (see table 1). Our approach included critical examination and reflection on the role of the researcher in data collection and analysis. In meetings with the research team, we reflected on our biases, assumptions, structural differences in relation to the research participants, and influence on the research process, which deepened the engagement with the data. Finally, we presented the content of our themes as shared meanings built around a central organizing concept, rather than as topic summaries. The concept of themes as shared meanings can be relevant to both codebook and reflexive approaches, although it is more common in reflexive TA approaches (Braun and Clarke 2022).

A potential risk associated with blended approaches is the proliferation of poorly grounded or underdeveloped TA approaches, such as combining methods that do not align with the researcher’s way of thinking (e.g., using intercoder reliability in analyses that rely on a subjective understanding of reality). Without a clear rationale, this can lead to research that lacks depth. Other common pitfalls of TA more broadly include not interpreting the data (i.e., only describing it), identifying themes as the end product of analysis without further contribution to the literature, and not connecting themes to each other (Staller 2015; Thorne 2020). By carefully considering the strengths and limitations of both singular and blended TA approaches, researchers can choose the most appropriate approach for their research.

In addition, our case study had several strengths and limitations. Strengths included prolonged engagement with the data, team meetings in which we reflected on our research, maintaining an audit trail of our analysis, researcher triangulation, and conducting a negative case analysis. Limitations included the (1) imbalance between the family approach and the comparison group in terms of sample size, (2) slightly different composition of the two groups in terms of demographic, family, and criminal background factors, (3) partially prevalent COVID-19 limitations restricting several modes of family contact during imprisonment, and (4) exclusive focus on fathers’ perspectives, excluding children and nonimprisoned caregivers’ perspectives. For a more detailed discussion of strengths and limitations of the case study, see Venema et al. (2024).

## Conclusion

In considering different approaches to TA, we argue that it is important to recognize that no single approach is inherently superior to another. More structured “orthodox” approaches provide a robust framework for novice TA researchers (Stevens 2023), which can be particularly valuable for validating and building on previous findings, thus ensuring continuity and coherence within the field of TA. The suitability of an approach depends on the researcher’s assumptions about reality, how knowledge is obtained, as well as the research objectives. The choice of a TA approach is also influenced by factors such as the research context, the nature of the data, whether the researcher is working in a team, and the size of the data corpus (see for example Parkinson et al. 2016; Byrne 2022). We emphasize that while this article and other guidelines for each approach to TA can guide researchers’ decisions, readers should not view them as rigid “rules.” Instead, it is important to consider why a particular approach is chosen and the implications of that decision for the research process and outcomes.

It is also important to note that, although blended approaches give researchers more freedom to adapt the structure of TA to their own needs, this freedom should not lead to poorly grounded or underdeveloped blended TA approaches (Braun and Clarke 2019, 2021). By carefully considering the strengths and limitations of both singular and blended TA approaches, researchers can choose the most appropriate approach for their specific research objectives and ensure the production of high-quality results.

To conclude, this paper has sought to provide a brief overview of the different forms of TA and guidance on how to conduct a blended approach to TA in practice. This contributes to the scholarly discussion on how blended approaches to TA can be thoughtfully conducted.

## Supplementary Material

nfaf033_Supplementary_Data
