# Supplementary material for: Using a Blended Approach to Thematic Analysis: A Case Study on Fatherhood and Imprisonment
Source: Public Opin Q. 2025 Aug 6;89(SI):716–33. doi: 10.1093/poq/nfaf033 (PMC12411909; doi:10.1093/poq/nfaf033)
Supplement: nfaf033_Supplementary_Data [file nfaf033_supplementary_data.pdf]

Title Page

Using a Blended Approach to Thematic Analysis: A Case Study on Fatherhood and Imprisonment

Author information:
1. Marieke Haan, Assistant Professor, Department of Sociology, Faculty of Behavioral and Social Sciences, University of Groningen, Groningen, The Netherlands.
2. Simon D. Venema, Researcher, Addiction Care Northern Netherlands, Groningen, the Netherlands, and Researcher, Research group of Addiction Science and Forensic Care, Hanze University of Applied Sciences, Groningen, the Netherlands.

Corresponding author contact information:
Marieke Haan
Marieke.haan@rug.nl

Supplementary Material:

S1. Main Results of Systematic Literature Review about Fatherhood in Prison.
Based on: Venema et al. 2022

Supplementary text and figure - S1

Main Results of Systematic Literature Review About Fatherhood in Prison Based on: Venema et al. 2022

Short Summary

A systematic review was conducted to synthesize the literature on paternal imprisonment and father-child relationships (FCRs). Four academic databases were searched for peer-reviewed studies. Thirty studies were identified. It was found that FCRs most often deteriorate due to paternal imprisonment, but sometimes remain stable or change positively. Four key factors were found to influence FCRs: (a) the quality of preprison FCRs, (b) the frequency and experience of father–child contact during imprisonment, (c) the child's primary caregivers' role in facilitating father–child contact, and (d) prison barriers for maintaining FCRs during imprisonment.

Main Findings

The subthemes identified in the 30 studies are thematically organized in an integrated framework of FCRs in the context of paternal imprisonment displayed in Figure A1. The subthemes are placed within the four dimensions that constitute FCRs that were identified in our theoretical framework: father–child interactions, perceptions of father–child relationship quality, family context, and prison context. In the figure, the dashed lines and arrows indicate that different dimensions of the framework may overlap and mutually reinforce each other.

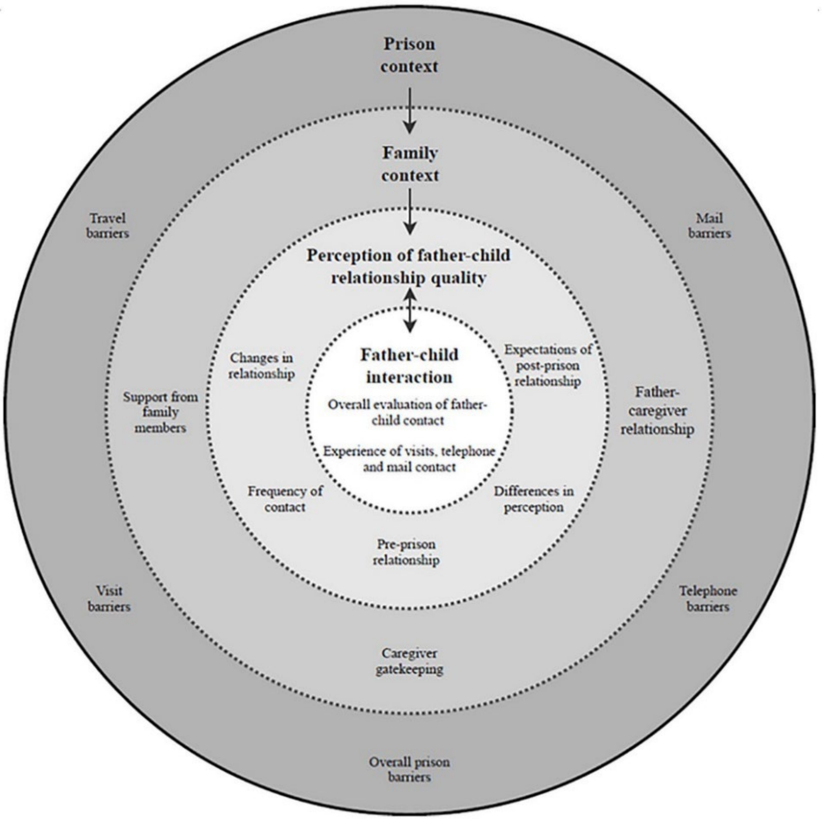

Figure S1. Integrated framework of father–child relationships and paternal imprisonment
